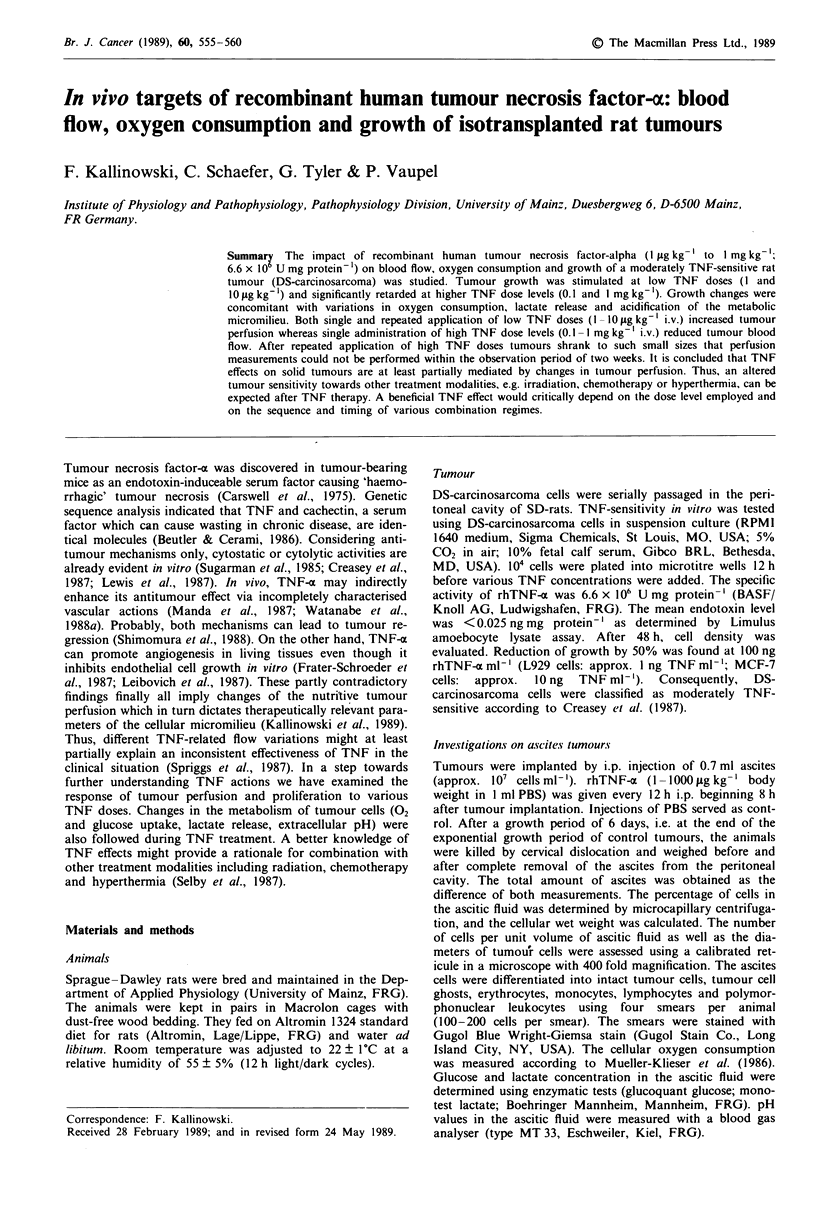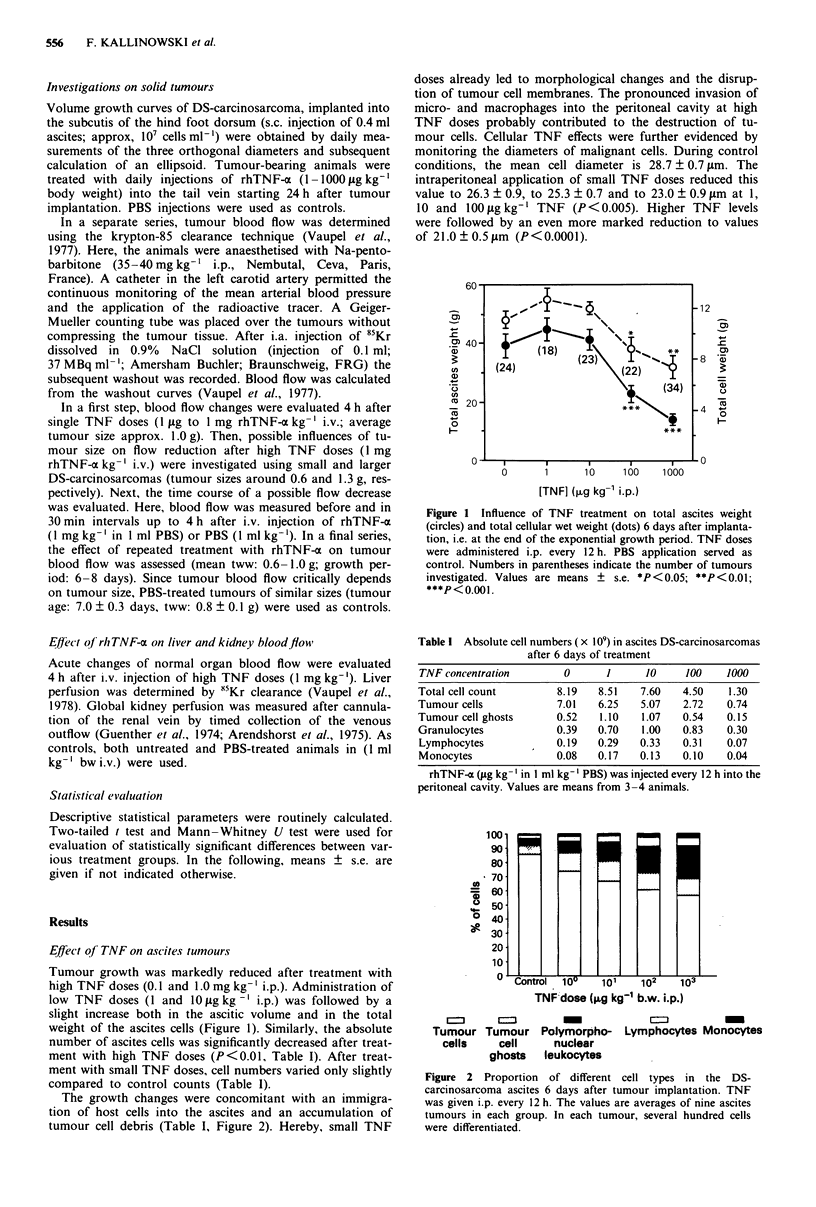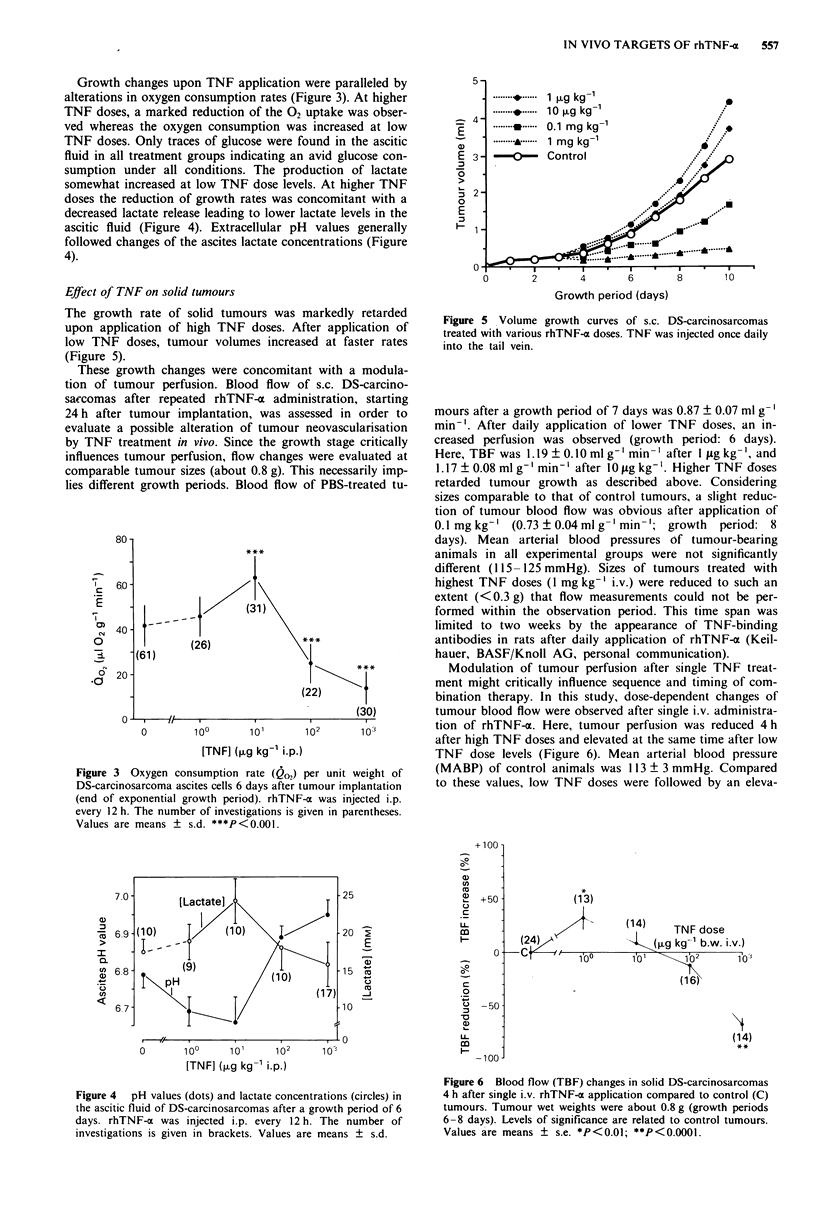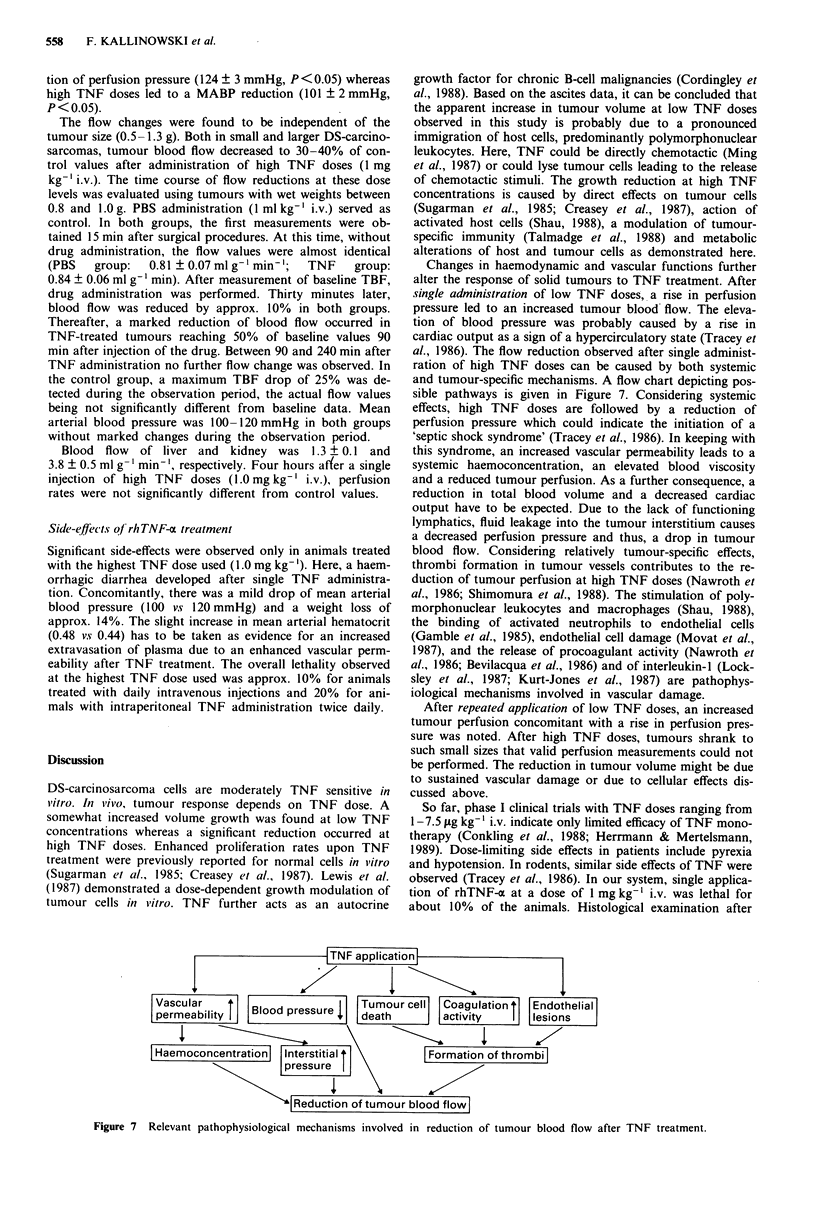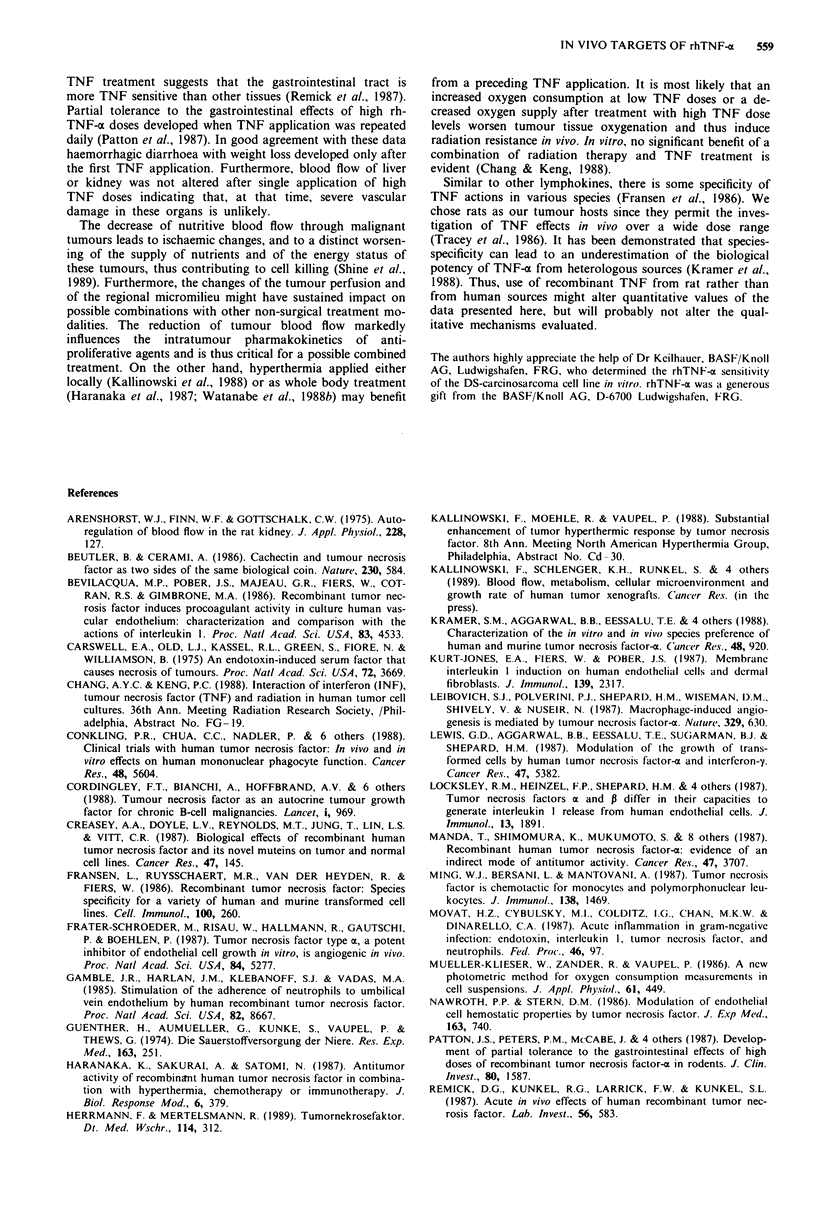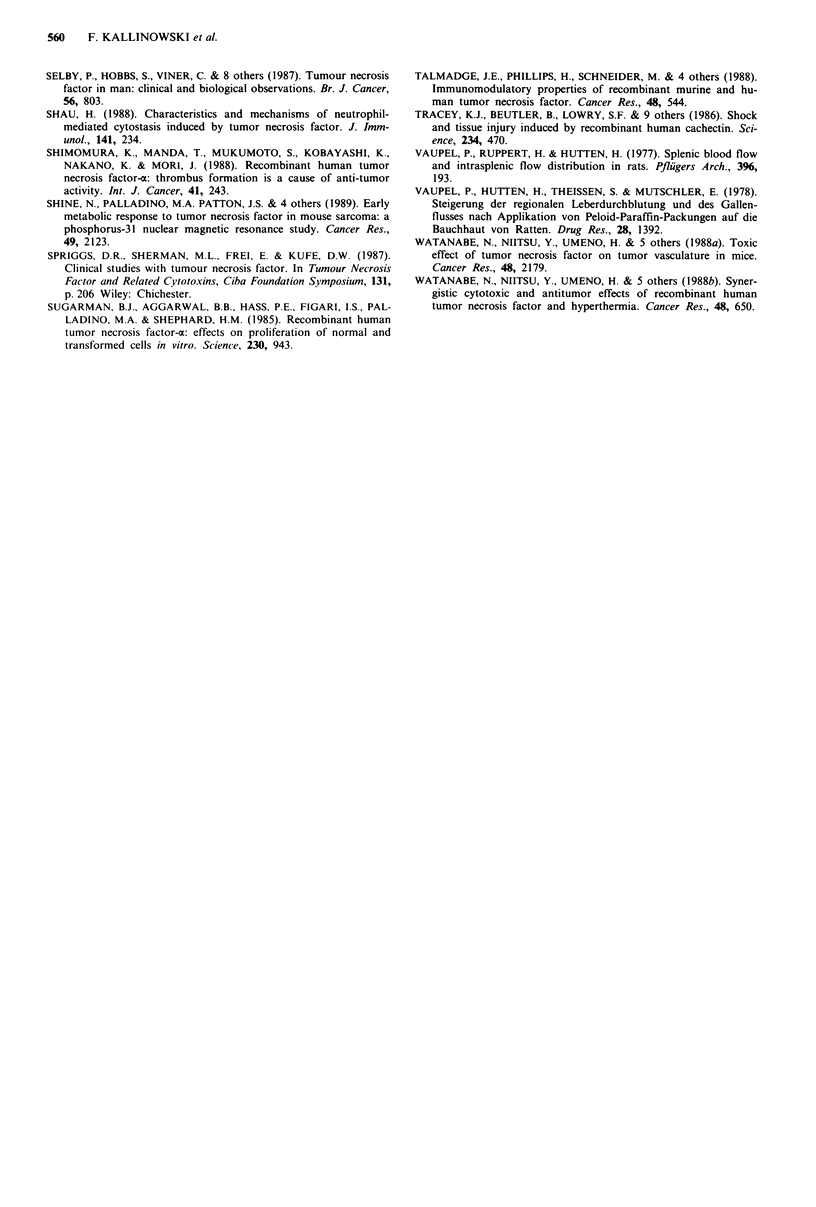# In vivo targets of recombinant human tumour necrosis factor-alpha: blood flow, oxygen consumption and growth of isotransplanted rat tumours.

**DOI:** 10.1038/bjc.1989.312

**Published:** 1989-10

**Authors:** F. Kallinowski, C. Schaefer, G. Tyler, P. Vaupel

**Affiliations:** Pathophysiology Division, University of Mainz, FR Germany.

## Abstract

The impact of recombinant human tumour necrosis factor-alpha (1 microgram kg-1 to 1 mg kg-1; 6.6 x 10(6) U mg protein-1) on blood flow, oxygen consumption and growth of a moderately TNF-sensitive rat tumour (DS-carcinosarcoma) was studied. Tumour growth was stimulated at low TNF doses (1 and 10 micrograms kg-1) and significantly retarded at higher TNF dose levels (0.1 and 1 mg kg-1). Growth changes were concomitant with variations in oxygen consumption, lactate release and acidification of the metabolic micromilieu. Both single and repeated application of low TNF doses (1-10 micrograms kg-1 i.v.) increased tumour perfusion whereas single administration of high TNF dose levels (0.1-1 mg kg-1 i.v.) reduced tumour blood flow. After repeated application of high TNF doses tumours shrank to such small sizes that perfusion measurements could not be performed within the observation period of two weeks. It is concluded that TNF effects on solid tumours are at least partially mediated by changes in tumour perfusion. Thus, an altered tumour sensitivity towards other treatment modalities, e.g. irradiation, chemotherapy or hyperthermia, can be expected after TNF therapy. A beneficial TNF effect would critically depend on the dose level employed and on the sequence and timing of various combination regimes.


Br. J. Cancer (1989), 60, 555–560 © The Macmillan Press Ltd., 1989

### *In vivo* targets of recombinant human tumour necrosis factor-α: blood flow, oxygen consumption and growth of isotransplanted rat tumours

F. Kallinowski, C. Schaefer, G. Tyler & P. Vaupel

*Institute of Physiology and Pathophysiology, Pathophysiology Division, University of Mainz, Duesbergweg 6, D-6500 Mainz, FR Germany.*

**Summary** The impact of recombinant human tumour necrosis factor-alpha ($1\,\mu g\,kg^{-1}$ to $1\,mg\,kg^{-1}$; $6.6 \times 10^6$ U mg protein$^{-1}$) on blood flow, oxygen consumption and growth of a moderately TNF-sensitive rat tumour (DS-carcinosarcoma) was studied. Tumour growth was stimulated at low TNF doses (1 and $10\,\mu g\,kg^{-1}$) and significantly retarded at higher TNF dose levels (0.1 and $1\,mg\,kg^{-1}$). Growth changes were concomitant with variations in oxygen consumption, lactate release and acidification of the metabolic micromilieu. Both single and repeated application of low TNF doses ($1-10\,\mu g\,kg^{-1}$ i.v.) increased tumour perfusion whereas single administration of high TNF dose levels ($0.1-1\,mg\,kg^{-1}$ i.v.) reduced tumour blood flow. After repeated application of high TNF doses tumours shrank to such small sizes that perfusion measurements could not be performed within the observation period of two weeks. It is concluded that TNF effects on solid tumours are at least partially mediated by changes in tumour perfusion. Thus, an altered tumour sensitivity towards other treatment modalities, e.g. irradiation, chemotherapy or hyperthermia, can be expected after TNF therapy. A beneficial TNF effect would critically depend on the dose level employed and on the sequence and timing of various combination regimes.

Tumour necrosis factor-α was discovered in tumour-bearing mice as an endotoxin-induceable serum factor causing 'haemorrhagic' tumour necrosis (Carswell *et al*., 1975). Genetic sequence analysis indicated that TNF and cachectin, a serum factor which can cause wasting in chronic disease, are identical molecules (Beutler & Cerami, 1986). Considering anti-tumour mechanisms only, cytostatic or cytolytic activities are already evident *in vitro* (Sugarman *et al*., 1985; Creasey *et al*., 1987; Lewis *et al*., 1987). *In vivo*, TNF-α may indirectly enhance its antitumour effect via incompletely characterised vascular actions (Manda *et al*., 1987; Watanabe *et al*., 1988*a*). Probably, both mechanisms can lead to tumour regression (Shimomura *et al*., 1988). On the other hand, TNF-α can promote angiogenesis in living tissues even though it inhibits endothelial cell growth *in vitro* (Frater-Schroeder *et al*., 1987; Leibovich *et al*., 1987). These partly contradictory findings finally all imply changes of the nutritive tumour perfusion which in turn dictates therapeutically relevant parameters of the cellular micromilieu (Kallinowski *et al*., 1989). Thus, different TNF-related flow variations might at least partially explain an inconsistent effectiveness of TNF in the clinical situation (Spriggs *et al*., 1987). In a step towards further understanding TNF actions we have examined the response of tumour perfusion and proliferation to various TNF doses. Changes in the metabolism of tumour cells ($O_2$ and glucose uptake, lactate release, extracellular pH) were also followed during TNF treatment. A better knowledge of TNF effects might provide a rationale for combination with other treatment modalities including radiation, chemotherapy and hyperthermia (Selby *et al*., 1987).

### Materials and methods

### *Animals*

Sprague–Dawley rats were bred and maintained in the Department of Applied Physiology (University of Mainz, FRG). The animals were kept in pairs in Macrolon cages with dust-free wood bedding. They fed on Altromin 1324 standard diet for rats (Altromin, Lage/Lippe, FRG) and water *ad libitum*. Room temperature was adjusted to $22 \pm 1°C$ at a relative humidity of $55 \pm 5\%$ (12 h light/dark cycles).

Correspondence: F. Kallinowski.
Received 28 February 1989; and in revised form 24 May 1989.

### *Tumour*

DS-carcinosarcoma cells were serially passaged in the peritoneal cavity of SD-rats. TNF-sensitivity *in vitro* was tested using DS-carcinosarcoma cells in suspension culture (RPMI 1640 medium, Sigma Chemicals, St Louis, MO, USA; 5% $CO_2$ in air; 10% fetal calf serum, Gibco BRL, Bethesda, MD, USA). $10^4$ cells were plated into microtitre wells 12 h before various TNF concentrations were added. The specific activity of rhTNF-α was $6.6 \times 10^6$ U mg protein$^{-1}$ (BASF/Knoll AG, Ludwigshafen, FRG). The mean endotoxin level was $<0.025$ ng mg protein$^{-1}$ as determined by Limulus amoebocyte lysate assay. After 48 h, cell density was evaluated. Reduction of growth by 50% was found at 100 ng rhTNF-α ml$^{-1}$ (L929 cells: approx. 1 ng TNF ml$^{-1}$; MCF-7 cells: approx. 10 ng TNF ml$^{-1}$). Consequently, DS-carcinosarcoma cells were classified as moderately TNF-sensitive according to Creasey *et al*. (1987).

### *Investigations on ascites tumours*

Tumours were implanted by i.p. injection of 0.7 ml ascites (approx. $10^7$ cells ml$^{-1}$). rhTNF-α ($1-1000\,\mu g\,kg^{-1}$ body weight in 1 ml PBS) was given every 12 h i.p. beginning 8 h after tumour implantation. Injections of PBS served as control. After a growth period of 6 days, i.e. at the end of the exponential growth period of control tumours, the animals were killed by cervical dislocation and weighed before and after complete removal of the ascites from the peritoneal cavity. The total amount of ascites was obtained as the difference of both measurements. The percentage of cells in the ascitic fluid was determined by microcapillary centrifugation, and the cellular wet weight was calculated. The number of cells per unit volume of ascitic fluid as well as the diameters of tumour cells were assessed using a calibrated reticule in a microscope with 400 fold magnification. The ascites cells were differentiated into intact tumour cells, tumour cell ghosts, erythrocytes, monocytes, lymphocytes and polymorphonuclear leukocytes using four smears per animal (100–200 cells per smear). The smears were stained with Gugol Blue Wright-Giemsa stain (Gugol Stain Co., Long Island City, NY, USA). The cellular oxygen consumption was measured according to Mueller-Klieser *et al*. (1986). Glucose and lactate concentration in the ascitic fluid were determined using enzymatic tests (glucoquant glucose; monotest lactate; Boehringer Mannheim, Mannheim, FRG). pH values in the ascitic fluid were measured with a blood gas analyser (type MT 33, Eschweiler, Kiel, FRG).

*Investigations on solid tumours*

Volume growth curves of DS-carcinosarcoma, implanted into the subcutis of the hind foot dorsum (s.c. injection of 0.4 ml ascites; approx, $10^7$ cells $ml^{-1}$) were obtained by daily measurements of the three orthogonal diameters and subsequent calculation of an ellipsoid. Tumour-bearing animals were treated with daily injections of rhTNF-α (1–1000 µg $kg^{-1}$ body weight) into the tail vein starting 24 h after tumour implantation. PBS injections were used as controls.

In a separate series, tumour blood flow was determined using the krypton-85 clearance technique (Vaupel *et al.*, 1977). Here, the animals were anaesthetised with Na-pentobarbitone (35–40 mg $kg^{-1}$ i.p., Nembutal, Ceva, Paris, France). A catheter in the left carotid artery permitted the continuous monitoring of the mean arterial blood pressure and the application of the radioactive tracer. A Geiger-Mueller counting tube was placed over the tumours without compressing the tumour tissue. After i.a. injection of $^{85}Kr$ dissolved in 0.9% NaCl solution (injection of 0.1 ml; 37 MBq $ml^{-1}$; Amersham Buchler; Braunschweig, FRG) the subsequent washout was recorded. Blood flow was calculated from the washout curves (Vaupel *et al.*, 1977).

In a first step, blood flow changes were evaluated 4 h after single TNF doses (1 µg to 1 mg rhTNF-α $kg^{-1}$ i.v.; average tumour size approx. 1.0 g). Then, possible influences of tumour size on flow reduction after high TNF doses (1 mg rhTNF-α $kg^{-1}$ i.v.) were investigated using small and larger DS-carcinosarcomas (tumour sizes around 0.6 and 1.3 g, respectively). Next, the time course of a possible flow decrease was evaluated. Here, blood flow was measured before and in 30 min intervals up to 4 h after i.v. injection of rhTNF-α (1 mg $kg^{-1}$ in 1 ml PBS) or PBS (1 ml $kg^{-1}$). In a final series, the effect of repeated treatment with rhTNF-α on tumour blood flow was assessed (mean tww: 0.6–1.0 g; growth period: 6–8 days). Since tumour blood flow critically depends on tumour size, PBS-treated tumours of similar sizes (tumour age: 7.0 ± 0.3 days, tww: 0.8 ± 0.1 g) were used as controls.

*Effect of rhTNF-α on liver and kidney blood flow*

Acute changes of normal organ blood flow were evaluated 4 h after i.v. injection of high TNF doses (1 mg $kg^{-1}$). Liver perfusion was determined by $^{85}Kr$ clearance (Vaupel *et al.*, 1978). Global kidney perfusion was measured after cannulation of the renal vein by timed collection of the venous outflow (Guenther *et al.*, 1974; Arendshorst *et al.*, 1975). As controls, both untreated and PBS-treated animals in (1 ml $kg^{-1}$ bw i.v.) were used.

*Statistical evaluation*

Descriptive statistical parameters were routinely calculated. Two-tailed *t* test and Mann–Whitney *U* test were used for evaluation of statistically significant differences between various treatment groups. In the following, means ± s.e. are given if not indicated otherwise.

### Results

*Effect of TNF on ascites tumours*

Tumour growth was markedly reduced after treatment with high TNF doses (0.1 and 1.0 mg $kg^{-1}$ i.p.). Administration of low TNF doses (1 and 10 µg $kg^{-1}$ i.p.) was followed by a slight increase both in the ascitic volume and in the total weight of the ascites cells (Figure 1). Similarly, the absolute number of ascites cells was significantly decreased after treatment with high TNF doses ($P<0.01$, Table I). After treatment with small TNF doses, cell numbers varied only slightly compared to control counts (Table I).

The growth changes were concomitant with an immigration of host cells into the ascites and an accumulation of tumour cell debris (Table I, Figure 2). Hereby, small TNF doses already led to morphological changes and the disruption of tumour cell membranes. The pronounced invasion of micro- and macrophages into the peritoneal cavity at high TNF doses probably contributed to the destruction of tumour cells. Cellular TNF effects were further evidenced by monitoring the diameters of malignant cells. During control conditions, the mean cell diameter is 28.7 ± 0.7 µm. The intraperitoneal application of small TNF doses reduced this value to 26.3 ± 0.9, to 25.3 ± 0.7 and to 23.0 ± 0.9 µm at 1, 10 and 100 µg $kg^{-1}$ TNF ($P<0.005$). Higher TNF levels were followed by an even more marked reduction to values of 21.0 ± 0.5 µm ($P<0.0001$).

**Figure 1** Influence of TNF treatment on total ascites weight (circles) and total cellular wet weight (dots) 6 days after implantation, i.e. at the end of the exponential growth period. TNF doses were administered i.p. every 12 h. PBS application served as control. Numbers in parentheses indicate the number of tumours investigated. Values are means ± s.e. *$P<0.05$; **$P<0.01$; ***$P<0.001$.

**Table I** Absolute cell numbers ( × $10^9$) in ascites DS-carcinosarcomas after 6 days of treatment

| *TNF concentration* | *0* | *1* | *10* | *100* | *1000* |
|---|---|---|---|---|---|
| Total cell count | 8.19 | 8.51 | 7.60 | 4.50 | 1.30 |
| Tumour cells | 7.01 | 6.25 | 5.07 | 2.72 | 0.74 |
| Tumour cell ghosts | 0.52 | 1.10 | 1.07 | 0.54 | 0.15 |
| Granulocytes | 0.39 | 0.70 | 1.00 | 0.83 | 0.30 |
| Lymphocytes | 0.19 | 0.29 | 0.33 | 0.31 | 0.07 |
| Monocytes | 0.08 | 0.17 | 0.13 | 0.10 | 0.04 |

rhTNF-α (µg $kg^{-1}$ in 1 ml $kg^{-1}$ PBS) was injected every 12 h into the peritoneal cavity. Values are means from 3–4 animals.

**Figure 2** Proportion of different cell types in the DS-carcinosarcoma ascites 6 days after tumour implantation. TNF was given i.p. every 12 h. The values are averages of nine ascites tumours in each group. In each tumour, several hundred cells were differentiated.

Growth changes upon TNF application were paralleled by alterations in oxygen consumption rates (Figure 3). At higher TNF doses, a marked reduction of the $O_2$ uptake was observed whereas the oxygen consumption was increased at low TNF doses. Only traces of glucose were found in the ascitic fluid in all treatment groups indicating an avid glucose consumption under all conditions. The production of lactate somewhat increased at low TNF dose levels. At higher TNF doses the reduction of growth rates was concomitant with a decreased lactate release leading to lower lactate levels in the ascitic fluid (Figure 4). Extracellular pH values generally followed changes of the ascites lactate concentrations (Figure 4).

*Effect of TNF on solid tumours*

The growth rate of solid tumours was markedly retarded upon application of high TNF doses. After application of low TNF doses, tumour volumes increased at faster rates (Figure 5).

These growth changes were concomitant with a modulation of tumour perfusion. Blood flow of s.c. DS-carcinosarcomas after repeated rhTNF-α administration, starting 24 h after tumour implantation, was assessed in order to evaluate a possible alteration of tumour neovascularisation by TNF treatment *in vivo*. Since the growth stage critically influences tumour perfusion, flow changes were evaluated at comparable tumour sizes (about 0.8 g). This necessarily implies different growth periods. Blood flow of PBS-treated tumours after a growth period of 7 days was 0.87 ± 0.07 ml $g^{-1}$ $min^{-1}$. After daily application of lower TNF doses, an increased perfusion was observed (growth period: 6 days). Here, TBF was 1.19 ± 0.10 ml $g^{-1}$ $min^{-1}$ after 1 μg $kg^{-1}$, and 1.17 ± 0.08 ml $g^{-1}$ $min^{-1}$ after 10 μg $kg^{-1}$. Higher TNF doses retarded tumour growth as described above. Considering sizes comparable to that of control tumours, a slight reduction of tumour blood flow was obvious after application of 0.1 mg $kg^{-1}$ (0.73 ± 0.04 ml $g^{-1}$ $min^{-1}$; growth period: 8 days). Mean arterial blood pressures of tumour-bearing animals in all experimental groups were not significantly different (115–125 mmHg). Sizes of tumours treated with highest TNF doses (1 mg $kg^{-1}$ i.v.) were reduced to such an extent (<0.3 g) that flow measurements could not be performed within the observation period. This time span was limited to two weeks by the appearance of TNF-binding antibodies in rats after daily application of rhTNF-α (Keilhauer, BASF/Knoll AG, personal communication).

Modulation of tumour perfusion after single TNF treatment might critically influence sequence and timing of combination therapy. In this study, dose-dependent changes of tumour blood flow were observed after single i.v. administration of rhTNF-α. Here, tumour perfusion was reduced 4 h after high TNF doses and elevated at the same time after low TNF dose levels (Figure 6). Mean arterial blood pressure (MABP) of control animals was 113 ± 3 mmHg. Compared to these values, low TNF doses were followed by an eleva-

**Figure 3** Oxygen consumption rate ($\dot{Q}_{O_2}$) per unit weight of DS-carcinosarcoma ascites cells 6 days after tumour implantation (end of exponential growth period). rhTNF-α was injected i.p. every 12 h. The number of investigations is given in parentheses. Values are means ± s.d. ***$P<0.001$.

**Figure 4** pH values (dots) and lactate concentrations (circles) in the ascitic fluid of DS-carcinosarcomas after a growth period of 6 days. rhTNF-α was injected i.p. every 12 h. The number of investigations is given in brackets. Values are means ± s.d.

**Figure 5** Volume growth curves of s.c. DS-carcinosarcomas treated with various rhTNF-α doses. TNF was injected once daily into the tail vein.

**Figure 6** Blood flow (TBF) changes in solid DS-carcinosarcomas 4 h after single i.v. rhTNF-α application compared to control (C) tumours. Tumour wet weights were about 0.8 g (growth periods 6–8 days). Levels of significance are related to control tumours. Values are means ± s.e. *$P<0.01$; **$P<0.0001$.

tion of perfusion pressure (124 ± 3 mmHg, $P<0.05$) whereas high TNF doses led to a MABP reduction (101 ± 2 mmHg, $P<0.05$).

The flow changes were found to be independent of the tumour size (0.5–1.3 g). Both in small and larger DS-carcinosarcomas, tumour blood flow decreased to 30–40% of control values after administration of high TNF doses (1 mg $kg^{-1}$ i.v.). The time course of flow reductions at these dose levels was evaluated using tumours with wet weights between 0.8 and 1.0 g. PBS administration (1 ml $kg^{-1}$ i.v.) served as control. In both groups, the first measurements were obtained 15 min after surgical procedures. At this time, without drug administration, the flow values were almost identical (PBS group: 0.81 ± 0.07 ml $g^{-1}$ $min^{-1}$; TNF group: 0.84 ± 0.06 ml $g^{-1}$ min). After measurement of baseline TBF, drug administration was performed. Thirty minutes later, blood flow was reduced by approx. 10% in both groups. Thereafter, a marked reduction of blood flow occurred in TNF-treated tumours reaching 50% of baseline values 90 min after injection of the drug. Between 90 and 240 min after TNF administration no further flow change was observed. In the control group, a maximum TBF drop of 25% was detected during the observation period, the actual flow values being not significantly different from baseline data. Mean arterial blood pressure was 100–120 mmHg in both groups without marked changes during the observation period.

Blood flow of liver and kidney was 1.3 ± 0.1 and 3.8 ± 0.5 ml $g^{-1}$ $min^{-1}$, respectively. Four hours after a single injection of high TNF doses (1.0 mg $kg^{-1}$ i.v.), perfusion rates were not significantly different from control values.

### *Side-effects of rhTNF-α treatment*

Significant side-effects were observed only in animals treated with the highest TNF dose used (1.0 mg $kg^{-1}$). Here, a haemorrhagic diarrhea developed after single TNF administration. Concomitantly, there was a mild drop of mean arterial blood pressure (100 *vs* 120 mmHg) and a weight loss of approx. 14%. The slight increase in mean arterial hematocrit (0.48 *vs* 0.44) has to be taken as evidence for an increased extravasation of plasma due to an enhanced vascular permeability after TNF treatment. The overall lethality observed at the highest TNF dose used was approx. 10% for animals treated with daily intravenous injections and 20% for animals with intraperitoneal TNF administration twice daily.

### Discussion

DS-carcinosarcoma cells are moderately TNF sensitive *in vitro*. *In vivo*, tumour response depends on TNF dose. A somewhat increased volume growth was found at low TNF concentrations whereas a significant reduction occurred at high TNF doses. Enhanced proliferation rates upon TNF treatment were previously reported for normal cells *in vitro* (Sugarman *et al.*, 1985; Creasey *et al.*, 1987). Lewis *et al.* (1987) demonstrated a dose-dependent growth modulation of tumour cells *in vitro*. TNF further acts as an autocrine growth factor for chronic B-cell malignancies (Cordingley *et al.*, 1988). Based on the ascites data, it can be concluded that the apparent increase in tumour volume at low TNF doses observed in this study is probably due to a pronounced immigration of host cells, predominantly polymorphonuclear leukocytes. Here, TNF could be directly chemotactic (Ming *et al.*, 1987) or could lyse tumour cells leading to the release of chemotactic stimuli. The growth reduction at high TNF concentrations is caused by direct effects on tumour cells (Sugarman *et al.*, 1985; Creasey *et al.*, 1987), action of activated host cells (Shau, 1988), a modulation of tumour-specific immunity (Talmadge *et al.*, 1988) and metabolic alterations of host and tumour cells as demonstrated here.

Changes in haemodynamic and vascular functions further alter the response of solid tumours to TNF treatment. After *single administration* of low TNF doses, a rise in perfusion pressure led to an increased tumour blood flow. The elevation of blood pressure was probably caused by a rise in cardiac output as a sign of a hypercirculatory state (Tracey *et al.*, 1986). The flow reduction observed after single administration of high TNF doses can be caused by both systemic and tumour-specific mechanisms. A flow chart depicting possible pathways is given in Figure 7. Considering systemic effects, high TNF doses are followed by a reduction of perfusion pressure which could indicate the initiation of a 'septic shock syndrome' (Tracey *et al.*, 1986). In keeping with this syndrome, an increased vascular permeability leads to a systemic haemoconcentration, an elevated blood viscosity and a reduced tumour perfusion. As a further consequence, a reduction in total blood volume and a decreased cardiac output have to be expected. Due to the lack of functioning lymphatics, fluid leakage into the tumour interstitium causes a decreased perfusion pressure and thus, a drop in tumour blood flow. Considering relatively tumour-specific effects, thrombi formation in tumour vessels contributes to the reduction of tumour perfusion at high TNF doses (Nawroth *et al.*, 1986; Shimomura *et al.*, 1988). The stimulation of polymorphonuclear leukocytes and macrophages (Shau, 1988), the binding of activated neutrophils to endothelial cells (Gamble *et al.*, 1985), endothelial cell damage (Movat *et al.*, 1987), and the release of procoagulant activity (Nawroth *et al.*, 1986; Bevilacqua *et al.*, 1986) and of interleukin-1 (Locksley *et al.*, 1987; Kurt-Jones *et al.*, 1987) are pathophysiological mechanisms involved in vascular damage.

After *repeated application* of low TNF doses, an increased tumour perfusion concomitant with a rise in perfusion pressure was noted. After high TNF doses, tumours shrank to such small sizes that valid perfusion measurements could not be performed. The reduction in tumour volume might be due to sustained vascular damage or due to cellular effects discussed above.

So far, phase I clinical trials with TNF doses ranging from 1–7.5 μg $kg^{-1}$ i.v. indicate only limited efficacy of TNF monotherapy (Conkling *et al.*, 1988; Herrmann & Mertelsmann, 1989). Dose-limiting side effects in patients include pyrexia and hypotension. In rodents, similar side effects of TNF were observed (Tracey *et al.*, 1986). In our system, single application of rhTNF-α at a dose of 1 mg $kg^{-1}$ i.v. was lethal for about 10% of the animals. Histological examination after

**Figure 7** Relevant pathophysiological mechanisms involved in reduction of tumour blood flow after TNF treatment.

TNF treatment suggests that the gastrointestinal tract is more TNF sensitive than other tissues (Remick *et al.*, 1987). Partial tolerance to the gastrointestinal effects of high rhTNF-α doses developed when TNF application was repeated daily (Patton *et al.*, 1987). In good agreement with these data haemorrhagic diarrhoea with weight loss developed only after the first TNF application. Furthermore, blood flow of liver or kidney was not altered after single application of high TNF doses indicating that, at that time, severe vascular damage in these organs is unlikely.

The decrease of nutritive blood flow through malignant tumours leads to ischaemic changes, and to a distinct worsening of the supply of nutrients and of the energy status of these tumours, thus contributing to cell killing (Shine *et al.*, 1989). Furthermore, the changes of the tumour perfusion and of the regional micromilieu might have sustained impact on possible combinations with other non-surgical treatment modalities. The reduction of tumour blood flow markedly influences the intratumour pharmakokinetics of antiproliferative agents and is thus critical for a possible combined treatment. On the other hand, hyperthermia applied either locally (Kallinowski *et al.*, 1988) or as whole body treatment (Haranaka *et al.*, 1987; Watanabe *et al.*, 1988*b*) may benefit from a preceding TNF application. It is most likely that an increased oxygen consumption at low TNF doses or a decreased oxygen supply after treatment with high TNF dose levels worsen tumour tissue oxygenation and thus induce radiation resistance *in vivo*. *In vitro*, no significant benefit of a combination of radiation therapy and TNF treatment is evident (Chang & Keng, 1988).

Similar to other lymphokines, there is some specificity of TNF actions in various species (Fransen *et al.*, 1986). We chose rats as our tumour hosts since they permit the investigation of TNF effects *in vivo* over a wide dose range (Tracey *et al.*, 1986). It has been demonstrated that species-specificity can lead to an underestimation of the biological potency of TNF-α from heterologous sources (Kramer *et al.*, 1988). Thus, use of recombinant TNF from rat rather than from human sources might alter quantitative values of the data presented here, but will probably not alter the qualitative mechanisms evaluated.

The authors highly appreciate the help of Dr Keilhauer, BASF/Knoll AG, Ludwigshafen, FRG, who determined the rhTNF-α sensitivity of the DS-carcinosarcoma cell line *in vitro*. rhTNF-α was a generous gift from the BASF/Knoll AG, D-6700 Ludwigshafen, FRG.

### References

ARENSHORST, W.J., FINN, W.F. & GOTTSCHALK, C.W. (1975). Autoregulation of blood flow in the rat kidney. *J. Appl. Physiol.*, **228**, 127.

BEUTLER, B. & CERAMI, A. (1986). Cachectin and tumour necrosis factor as two sides of the same biological coin. *Nature*, **230**, 584.

BEVILACQUA, M.P., POBER, J.S., MAJEAU, G.R., FIERS, W., COTRAN, R.S. & GIMBRONE, M.A. (1986). Recombinant tumor necrosis factor induces procoagulant activity in culture human vascular endothelium: characterization and comparison with the actions of interleukin 1. *Proc. Natl Acad. Sci. USA*, **83**, 4533.

CARSWELL, E.A., OLD, L.J., KASSEL, R.L., GREEN, S., FIORE, N. & WILLIAMSON, B. (1975) An endotoxin-induced serum factor that causes necrosis of tumours. *Proc. Natl Acad. Sci. USA*, **72**, 3669.

CHANG, A.Y.C. & KENG, P.C. (1988). Interaction of interferon (INF), tumour necrosis factor (TNF) and radiation in human tumor cell cultures. 36th Ann. Meeting Radiation Research Society, /Philadelphia, Abstract No. FG-19.

CONKLING, P.R., CHUA, C.C., NADLER, P. & 6 others (1988). Clinical trials with human tumor necrosis factor: *In vivo* and *in vitro* effects on human mononuclear phagocyte function. *Cancer Res.*, **48**, 5604.

CORDINGLEY, F.T., BIANCHI, A., HOFFBRAND, A.V. & 6 others (1988). Tumour necrosis factor as an autocrine tumour growth factor for chronic B-cell malignancies. *Lancet*, **i**, 969.

CREASEY, A.A., DOYLE, L.V., REYNOLDS, M.T., JUNG, T., LIN, L.S. & VITT, C.R. (1987). Biological effects of recombinant human tumor necrosis factor and its novel muteins on tumor and normal cell lines. *Cancer Res.*, **47**, 145.

FRANSEN, L., RUYSSCHAERT, M.R., VAN DER HEYDEN, R. & FIERS, W. (1986). Recombinant tumor necrosis factor: Species specificity for a variety of human and murine transformed cell lines. *Cell. Immunol.*, **100**, 260.

FRATER-SCHROEDER, M., RISAU, W., HALLMANN, R., GAUTSCHI, P. & BOEHLEN, P. (1987). Tumor necrosis factor type α, a potent inhibitor of endothelial cell growth *in vitro*, is angiogenic *in vivo*. *Proc. Natl Acad. Sci. USA*, **84**, 5277.

GAMBLE, J.R., HARLAN, J.M., KLEBANOFF, S.J. & VADAS, M.A. (1985). Stimulation of the adherence of neutrophils to umbilical vein endothelium by human recombinant tumor necrosis factor. *Proc. Natl Acad. Sci. USA*, **82**, 8667.

GUENTHER, H., AUMUELLER, G., KUNKE, S., VAUPEL, P. & THEWS, G. (1974). Die Sauerstoffversorgung der Niere. *Res. Exp. Med.*, **163**, 251.

HARANAKA, K., SAKURAI, A. & SATOMI, N. (1987). Antitumor activity of recombinant human tumor necrosis factor in combination with hyperthermia, chemotherapy or immunotherapy. *J. Biol. Response Mod.*, **6**, 379.

HERRMANN, F. & MERTELSMANN, R. (1989). Tumornekrosefaktor. *Dt. Med. Wschr.*, **114**, 312.

KALLINOWSKI, F., MOEHLE, R. & VAUPEL, P. (1988). Substantial enhancement of tumor hyperthermic response by tumor necrosis factor. 8th Ann. Meeting North American Hyperthermia Group, Philadelphia, Abstract No. Cd-30.

KALLINOWSKI, F., SCHLENGER, K.H., RUNKEL, S. & 4 others (1989). Blood flow, metabolism, cellular microenvironment and growth rate of human tumor xenografts. *Cancer Res.* (in the press).

KRAMER, S.M., AGGARWAL, B.B., EESSALU, T.E. & 4 others (1988). Characterization of the *in vitro* and *in vivo* species preference of human and murine tumor necrosis factor-α. *Cancer Res.*, **48**, 920.

KURT-JONES, E.A., FIERS, W. & POBER, J.S. (1987). Membrane interleukin 1 induction on human endothelial cells and dermal fibroblasts. *J. Immunol.*, **139**, 2317.

LEIBOVICH, S.J., POLVERINI, P.J., SHEPARD, H.M., WISEMAN, D.M., SHIVELY, V. & NUSEIR, N. (1987). Macrophage-induced angiogenesis is mediated by tumour necrosis factor-α. *Nature*, **329**, 630.

LEWIS, G.D., AGGARWAL, B.B., EESSALU, T.E., SUGARMAN, B.J. & SHEPARD, H.M. (1987). Modulation of the growth of transformed cells by human tumor necrosis factor-α and interferon-γ. *Cancer Res.*, **47**, 5382.

LOCKSLEY, R.M., HEINZEL, F.P., SHEPARD, H.M. & 4 others (1987). Tumor necrosis factors α and β differ in their capacities to generate interleukin 1 release from human endothelial cells. *J. Immunol.*, **13**, 1891.

MANDA, T., SHIMOMURA, K., MUKUMOTO, S. & 8 others (1987). Recombinant human tumor necrosis factor-α: evidence of an indirect mode of antitumor activity. *Cancer Res.*, **47**, 3707.

MING, W.J., BERSANI, L. & MANTOVANI, A. (1987). Tumor necrosis factor is chemotactic for monocytes and polymorphonuclear leukocytes. *J. Immunol.*, **138**, 1469.

MOVAT, H.Z., CYBULSKY, M.I., COLDITZ, I.G., CHAN, M.K.W. & DINARELLO, C.A. (1987). Acute inflammation in gram-negative infection: endotoxin, interleukin 1, tumor necrosis factor, and neutrophils. *Fed. Proc.*, **46**, 97.

MUELLER-KLIESER, W., ZANDER, R. & VAUPEL, P. (1986). A new photometric method for oxygen consumption measurements in cell suspensions. *J. Appl. Physiol.*, **61**, 449.

NAWROTH, P.P. & STERN, D.M. (1986). Modulation of endothelial cell hemostatic properties by tumor necrosis factor. *J. Exp Med.*, **163**, 740.

PATTON, J.S., PETERS, P.M., McCABE, J. & 4 others (1987). Development of partial tolerance to the gastrointestinal effects of high doses of recombinant tumor necrosis factor-α in rodents. *J. Clin. Invest.*, **80**, 1587.

REMICK, D.G., KUNKEL, R.G., LARRICK, F.W. & KUNKEL, S.L. (1987). Acute *in vivo* effects of human recombinant tumor necrosis factor. *Lab. Invest.*, **56**, 583.

SELBY, P., HOBBS, S., VINER, C. & 8 others (1987). Tumour necrosis factor in man: clinical and biological observations. *Br. J. Cancer*, **56**, 803.

SHAU, H. (1988). Characteristics and mechanisms of neutrophil-mediated cytostasis induced by tumor necrosis factor. *J. Immunol.*, **141**, 234.

SHIMOMURA, K., MANDA, T., MUKUMOTO, S., KOBAYASHI, K., NAKANO, K. & MORI, J. (1988). Recombinant human tumor necrosis factor-α: thrombus formation is a cause of anti-tumor activity. *Int. J. Cancer*, **41**, 243.

SHINE, N., PALLADINO, M.A. PATTON, J.S. & 4 others (1989). Early metabolic response to tumor necrosis factor in mouse sarcoma: a phosphorus-31 nuclear magnetic resonance study. *Cancer Res.*, **49**, 2123.

SPRIGGS, D.R., SHERMAN, M.L., FREI, E. & KUFE, D.W. (1987). Clinical studies with tumour necrosis factor. In *Tumour Necrosis Factor and Related Cytotoxins, Ciba Foundation Symposium*, **131**, p. 206 Wiley: Chichester.

SUGARMAN, B.J., AGGARWAL, B.B., HASS, P.E., FIGARI, I.S., PALLADINO, M.A. & SHEPHARD, H.M. (1985). Recombinant human tumor necrosis factor-α: effects on proliferation of normal and transformed cells *in vitro*. *Science*, **230**, 943.

TALMADGE, J.E., PHILLIPS, H., SCHNEIDER, M. & 4 others (1988). Immunomodulatory properties of recombinant murine and human tumor necrosis factor. *Cancer Res.*, **48**, 544.

TRACEY, K.J., BEUTLER, B., LOWRY, S.F. & 9 others (1986). Shock and tissue injury induced by recombinant human cachectin. *Science*, **234**, 470.

VAUPEL, P., RUPPERT, H. & HUTTEN, H. (1977). Splenic blood flow and intrasplenic flow distribution in rats. *Pflügers Arch.*, **396**, 193.

VAUPEL, P., HUTTEN, H., THEISSEN, S. & MUTSCHLER, E. (1978). Steigerung der regionalen Leberdurchblutung und des Gallenflusses nach Applikation von Peloid-Paraffin-Packungen auf die Bauchhaut von Ratten. *Drug Res.*, **28**, 1392.

WATANABE, N., NIITSU, Y., UMENO, H. & 5 others (1988*a*). Toxic effect of tumor necrosis factor on tumor vasculature in mice. *Cancer Res.*, **48**, 2179.

WATANABE, N., NIITSU, Y., UMENO, H. & 5 others (1988*b*). Synergistic cytotoxic and antitumor effects of recombinant human tumor necrosis factor and hyperthermia. *Cancer Res.*, **48**, 650.